# Supplementary material for: Heat adaptation of phage T7 under an extended genetic code
Source: Virus Evol. 2021 Dec 1;7(2):veab100. doi: 10.1093/ve/veab100 (PMC8923235; doi:10.1093/ve/veab100)
Supplement: veab100_Supp [file veab100_supp.zip › Sequencing_Analysis.zip!/Sequencing_Analysis/A1/evidence/JC_SIDE_1_59.html]

BRESEQ :: Evidence


| New junction evidence | | | | | | | | | | | |
| --- | --- | --- | --- | --- | --- | --- | --- | --- | --- | --- | --- |
|  | | seq id | position | reads (cov) | reads (cov) | score | skew | freq | annotation | gene | product |
| \* | ? | NC\_001604.1 | = 13455 | 684 (0.220) | 103 (0.030) | 80/586 | 17.8 | 20.7% | intergenic (–/–) | *–/–* | –/– |
|  | ? | NC\_001604.1 | 14331 = | 121 (0.040) |  |  |  |  | intergenic (–/–) | *–/–* | –/– |
| Rejected: Coverage evenness skew score above cutoff. | | | | | | | | | | | |

Only 100 of 2281 total aligned reads displayed.  

`GGCTACATGGAATACAACAAGGAAACCGGATGGCTTGAACCATCAAGTTACTCAGGGGAAGAAGAGTCACACTCAGAGTCAACAGACTGGTCCAACGACACTGACTTCTGACAGGATTCTTGATGACTTTCCAGACGACTACGAGAAGTTTCGCTGGAGAGTCCCATTCTAATACGACTCACTAAAGGAGACACACCATGTTCAAACTGATTAAGAAGTTAGGCCAACTGCTGGTTCGTATGTACAACGTGGAAGCCAAGCGACTGAACGATGAGGCTCGTAAAGAGGCCACACAGTCACGCGCTCTGGCGATTCGCTCCAACGAACTGGCTGACAGTGCATCCACTAAAGTTACCGAGGCTGCCCGTGTGGCAAACCAAGCTCAACAGCTTTCCAAATTCTTTGAGTAATCAAACAGGAGAAACCATTATGTCTAACGTAGCTGAAACTATCCGTCTATCCGATACAGCTGACCAGTGGAACCGTCGAGTCCACATCAACGTTCGCAACGGTAAGGCGACTATGGTTTACCGCTGGAAGGACTCTAAGTCCTCTAAGAATCACACTCAGCGTATGACGTTGACAGATGAGCAAGCAC  >  NC_001604.1/13155‑13752`  

                                                                                                                                                                                                                                                                                                            `|`                                                                                                                                                                                                                                                                                                           
`ggCTACATGTAATACAACAAGGAAACCTGATGGCTTGAACCATCAAGTTACTCAGGGGAAGAAGAGTCACACTCAGAGTCAACAGACTGGTCCAACGACACTGACTTCTGACAGGATTCTTGATGACTTTCCAGACGACTACGAGAAGTTTCGCTGGAGAGTCCCATTCTAATACGACTCACTAAAGGAGACACACCATGTTCAAACTGATTAAGAAGTTAGGCCAACTGCTGGTTCGTATGTACAACGTGGAAGCCAAGCGACTGAACGATGAGGCTCGTAAAGAGGCCACACAGTCAcg                                                                                                                                                                                                                                                                                                           <  1:213685/301‑1 (MQ=255)`  
         `gAATACAACAAGGAAACCGGATGGCTTGAACCATCAAGTTACTCAGGGGAAGAAGAGTCACACTCAGAGTCAACAGACTGGTCCAACGACACTGACTTCTGACAGGATTCTTGATGACTTTCCAGACGACTACGAGAAGTTTCGCTGGAGAGTCCCATTCTAATACGACTCACTAAAGGAGACACACCATGTTCAAACTGATTAAGAAGTTAGGCCAACTGCTGGTTCGTATGTACAACGTGGAAGCCAAGCGACTGAACGATGAGGCTCGTAAAGATGCCACACAGTCACGCGCTCTGGc                                                                                                                                                                                                                                                                                                  >  1:199072/1‑301 (MQ=255)`  
         `gAATACAACAAGGAAACCGGATGGCTTGAACCATCAAGTTACTCAGGGGAAGAAGAGTCACACTCAGAGTCAACAGACTGGTCCAACGACACTGACTTCTGACAGGATTCTTGATGACTTTCCAGACGACTACGAGAAGTTTCGCTGGAGAGTCCCATTCTAATACGACTCACTAAAGGAGACACACCATGTTCAAACTGATTAAGAAGTTAGGCCAACTGCTGGTTCGTATGTACAACGTGGAAGCCAAGCGACTGAACGATGAGGCTCGTAAAGAGGCAACACAGTCAcgataatcaaa                                                                                                                                                                                                                                                                                                  >  1:171969/1‑292 (MQ=255)`  
           `aaagcaaaaGGAAACCAGATAGCTTGAACCCTCAGGTTACCCAGGGGAAAAAAGATCAGACTCATGGTCAATAAACTTGTCCAAAGGCACTTACTTCTGACAGGATTCTTGTTGACTTTCCAGACGACTACGAGAAGTTTCGCTGGAGAGTCCCATTCTAATACGACTCACTAAAGGAGACACACCATGTTCAAACTGATTAAGAAGTTAGGCCAACTGCTGGTTCGTATGTACAACGTGGAAGCCAAGCGACTGAACGATGAGGCTCGTAAAGAGGCCACACAGTCACGCGCTCTGGCGa                                                                                                                                                                                                                                                                                                <  1:194906/294‑1 (MQ=255)`  
                   `aGGAAACCGGATGGCTTGAACCATCAAGTTACTCAGGGGAAGAAGAGTCACACTCAGAGTCAACAGACTGGTCCAACTACACTGACTTCTGACAGGATTCTTGATGACTTTCCAGACGACTACGAGAAGTTTCGCTGGAGAGTCCCATTCTAATACGACTCACTAAAGGAGACACACCATGTTCAAACTGATTAAGAAGTTAGGCCAACTGCTGGTTCGTATGTACAACGTGGAAGCCAAGCGACTGAACGATGAGGCTCGTAAAGAGGCCACACAGTCACGCGCTCTGGCGATTCGCTcc                                                                                                                                                                                                                                                                                        <  1:273021/301‑1 (MQ=255)`  
                     `gAAACCGGATGGCTTGAACCATCAAGTTACTCAGTGGAAGAAGAGTCACACTCAGATTCAACAGACTGGTCCAACGACACTGACTTCTGACAGGATTCTTGATGACTTTCAAGACGACTACGAGAAGTTTCGCTGGAGAGTCCCATTCTAATACGACTCACTAAAGGAGACACACCATGTTCAAACTGATTAAGAAGTTAGGCCAACTGCTGGTTCGTATGTACAACGTGGAAGCCAAACAACTATACTAAGAGGATCCAAAAGACGCCACACAGTCACGCGCTCTGGCGATTccgtcact                                                                                                                                                                                                                                                                                      >  1:69321/1‑294 (MQ=255)`  
                                `gCTTGAACCATCCAGTTACTCAGGGGAAGAAGAGTCACACTCAGAGTCAACAGACTGGTCCCACTACACTGACTTCTGACAGGCTTCTTGATGACTTTCCAGACGACTACTCGAACTTTCGCTCGCGAGTCCCATTCTACTACGACTCACTAAAGGAGACACACCATGTTCAAACTGATTAAGCAGTTATGCCAACTGCTGGTTCGTATGTACAACGTGGAAGCCATGCGACTACACGATTAGGCTCTTAATGAGACCGCCCATTCACTCACTCTTGCGATTCCCTCCCACGCAccttttt                                                                                                                                                                                                                                                                           >  1:371323/1‑295 (MQ=255)`  
                                    `gAACCATCAAGTTACTCAGGGGAAGAAGAGTCACACTCAGAGTCAACAGACTTGTCCAACGACACTGACTTCTGACAGGATTCTTGATGACTTTCCAGACGACTACGAGAAGTTTCGCTGGAGAGTCCCATTCTAATACGACTCACTAAAGGAGACACACCATGTTCAAACTGATTAAGAAGTTAGGCCAACTGCTGGTTCGTATGTACAACGTGGAAGCCAAGCGACTGAACGATGAGGCTCGTAAAGAGGCCACACAGTCACGCGCTCTGGCGATTCGCTCCAACGAACTGGCTGACAg                                                                                                                                                                                                                                                                       <  1:126796/301‑1 (MQ=255)`  
                                          `tataagagacagggggAAGAAGAGTCACACTCAGAGTCAACAGACTGGTCCAACGACACTGACTTCTGACAGGATTCTTGATGACTTTACAGACGACTACGAGAAGTTTCGCTGGAGAGTCCCATTCTAATACGACTCACTAAAGGAGACACACCATGTTCAAACTGATTAAGAAGTTAGGCCAACTGCTGGTTCGTATGTACAACGTGGAAGCCAAGCGACTGAACGATGAGGCTCGTAAAGAGGCCACACAGTCACGCGCTCTGGCGATTCGCTCCAACGAACTGGCTGACAGTGCATc                                                                                                                                                                                                                                                                 <  1:242024/289‑1 (MQ=255)`  
                                             `ctcggagaagtgtaaaagggacagcagtCAGAGTCAACAGACTGGTCAAACGACACTGACTTCGGACAGGGTTCTTGATGACTTACCAGACGACTACGAGAAGTTTCGCTGGAGAGTCCCATTCTAATACGACTCACTAAAGGAGACACACCATGTTCAAACTGATTAAGAAGTTAGGCCAACTGCTGGTTCGTATGTACAACGTGGAAGCCAAGCGACTGAACGATGAGGCTCGTAAAGAGGCCACACAGTCACGCGCTCTGGCGATTAGCTCCAACGAACTGGCTGACAGTGCATCCAc                                                                                                                                                                                                                                                              <  1:470832/274‑1 (MQ=255)`  
                                             `aGTTACTCAGGGGAAGAAGAGTCACACTCAGAGTCAACAGACTGGTCCAACGACACTGACTTCTGACAGGATTCTTGATGACTTTCCAGACGACTACGAGAAGTTTCGCTGGAGAGTCCCATTCTAATACGACTCACTAAAGGAGACACACCATGTTCAAACTGATTAAGAAGTTAGGCCAACTGCTGGTTCGTATGTACAACGTGGAAGCCAAGCGAATGAACGATGAGGCTCGTAAAGAGGCCACAAAGTCACGCGCTCTGGCGATTCGCTCCAACGAACTGGCTGAAAGTGAATCCAc                                                                                                                                                                                                                                                              >  1:43635/1‑301 (MQ=255)`  
                                                   `ccAGGAGAAGAACAGTCACACTCAGAGTCAACAGACTTGTCCAACGCCACTGACTTCTTACAGGATTCTTGATGACGTTCCAGACGACTAGGAGAAGTTTCGCTTGAGAGTCCCATTCTAATACGACTCACTAAAGGAGACACACCATGTTCAAACTGATTAAGAAGTTAGGCCAACTGCTGGTTCGTATGTACAACGGGGAAGCCAAGCGACTGAACGATGAGGCTCGTAAAGAGGCCACACAGTCACGCGCTCTGGCGATTCGCTCCAACGAACTGGCTGACAGTGCATCCACTAAAGt                                                                                                                                                                                                                                                        <  1:94198/300‑1 (MQ=255)`  
                                                           `agaagaGTCACACTCAGAGTCAACAGACTGGTCCAACGACACTGACTTCTGACAGGATTCTTGATGACTTTCCAGACGACTACGAGAAGTTTCGCTGGAGAGTCCCATTCTAATACGACTCACTAAAGGAGACACACCATGTTCAAACTGATTAAGAAGTTAGGCCAACTGCTGGTTCGTATGTACAACGTGGAAGCCAAGCGACTGAACGATGAGGCTCGTAAAGAGGCCACACAGTCACGCGCTCTGGCGATTCGCTCCAACGAACTGGCTGACAGTGCATCCACTAAAGTTACCGAgg                                                                                                                                                                                                                                                <  1:389896/301‑1 (MQ=255)`  
                                                                `agTCACACTCAGAGTCAACAGACTGGTCCAACGACACTGACTTCTGACAGGATTCTTGATGACTTTCCAGACGACTACGAGAAGTTTCGCTGGAGAGTCCCATTCTAATACGACTCACTAAAGGAGACACACCATGTTCAAACTGATTAAGAAGTTAGGCCAACTGCTGGTTCGTATGTACAACGTGGAAGCCAAGCGACTGAACGATGAGGCTCGTAAAGAGGCCACACAGTCACGCGCTCTGGCGATTCGCTCCAACGAACTGGCTGACAGTGCATCCACTAAAGTTACCGAGGCTGcc                                                                                                                                                                                                                                           <  1:163542/301‑1 (MQ=255)`  
                                                                 `gTCACACTCAGAGTCAACAGACTGGTCCAACGACACTGACTTCTGACAGGATTCTTGATGACTTTCCAGACGACTACGAGAAGTTTCGCTGTAGAGTCCCATTCTAATACGACTCACTAAAGGAGACACACCATGTTCAAACTGATTAAGAAGTTAGGCCAACTGCTGGTTCGTATGTACAACGTGGAAGCCAAGCGACTGAACGATGAGGCTCGTAAAGCGGCCAAACAGTCCCCCACTCTGGCGATTCGCTCCAACGAACTGACTGACAGTGCATCCAATAACGTTACCggacctgacc                                                                                                                                                                                                                                          >  1:299184/1‑292 (MQ=255)`  
                                                                     `cacTCAGAGTCAACAGACTGGTCCAACGACACTGACTTCTGACAGGATTCTTGATGACTTTCCAGACGACTACGAGAAGTTTCGCTGGAGAGTCCCATTCTAATACGACTCACTAAAGGAGACACACCATGTTCAAACTGATTAAGAAGTTAGGCCAACTGCTGGTTCGTATGTACAACGTGGAAGCCAAGCGACTGAACGATGAGGCTCGTAAAGAGGCCACACAGTCACGCGCTCTGGCGATTCGCTCCAACGAACTGGCTGACAGTGCATCCACTAAAGTTACCGAGGCTGCCCgttt                                                                                                                                                                                                                                      >  1:91036/1‑299 (MQ=255)`  
                                                                       `cTCAGAGTCAACAGACTGGTCCAACGACACTGACTTCTGACAGGATTCTTGATGACTTTCCAGACGACTACGAGAAGTTTCGCTGGAGAGTCCCATTCTAATACGACTCACTAAAGGAGACACACCATGTTCAAACTGATTAAGAAGGTAGGCCAACTGCTGGTTCGTATGTACAACGTGGAAGCCAAGCGACTGAACGATGAGGCTCGTAAAGAGGCCTAACAGTCACGCGCTCTGGCGATTCGCTCCAACGAACTTGCTGACAGTGCATCCACCAAAGTTACCGAGGCTGCCCGTGTgg                                                                                                                                                                                                                                    >  1:226258/1‑301 (MQ=255)`  
                                                                        `agacagTCAACAGACTGGTCCAACGACACTGACTTCTGACAGGATTCTTGATGACTTTCCAGACGACTACGAGAAGTTTCGCTGGAGAGTCCCATTCTAATACGACTCACTAAAGGAGACACACCATGTTCAAACTGATTAAGAAGTTAGGCCAACTGCTGGTTCGTATGTACAACGTGGAAGCCAAGCGACTGAACGATGAGGCTCGTAAAGAGGCCACACAGTCACGCGCTCTGGCGATTCGCTCCAACGAACTGGCTGACAGTGCATCCACTAAAGTTACCGAGGATGCCCGTGTGGc                                                                                                                                                                                                                                   <  1:185379/297‑1 (MQ=255)`  
                                                                           `gagTCAACAGACTGGTCCAACGACACTGACTTCTGACAGGATTCTTGATGACTTTCCAGACGACTACGAGAAGTTTCGCTGGAGAGTCCCATTCTAATACGACTCACTAAAGGAGACACACCATGTTCAAACTGATTAAGAAGTTAGGCCAACTGCTGGTTCGTATGTACAACGTGGAAGCCAAGCGACTTAACGATGAGGCTCGTAAAGAGGCCACACAGTCACGCGCTCTGGCGATTCGCTCCAACGAACTGGCTGACAGTGCATCCACTaacgctgtctcttatacacatctccgagc                                                                                                                                                                                                                                >  1:217842/1‑274 (MQ=255)`  
                                                                             `gTCAACATACTGGTCCAACGACACTGACTTCTGACAGGGTTCTTGATGACTTTCCAGACTACTACGAGAAGTTTCGCTGGATGTTCCCATTCTAATACGACTCACTAAAGGAGACACACCATGTTCAAACTGATTAAGAAGTTAGGCCAACTGCTGGTTCGAATGTACAACGTGGAAGCTAAGCGACTGAACGATGATGCTCGTAAATAGGCCACACAGTCACGCTCACTGGCGATTCGCTCCAACGAACTTGCTGACAGTTCATCCACTACAGTTACCGAGGCTTCCCGTGTGGcgaccc                                                                                                                                                                                                                              >  1:190686/1‑296 (MQ=255)`  
                                                                               `cAACAGACTGGTCCAACGACACTGACTTCTGACAGGATTCTTGATGACTTTCCAGACGACTACGAGAAGTTTCGCTTGAGAGTATCATTCTAATACGACTCAATAAAGGAGACACACCAAGTTCAAAATGATTAAGAAGTTACGACAACTGCTGGTTCGAATGTACAACGTGGAAGCCAAGCGAATGAACGATGAGGCTCGTAAAGAGCTCACACACTCACGCGCTCTGTCGACTCGCTCCAACGAACTGGCTCACACTTCATCCACTAAAGTTACCCATGCTGTCCGTGTGGccacccca                                                                                                                                                                                                                            >  1:419740/1‑294 (MQ=255)`  
                                                                                      `agtataagagacagcTGACTTCTGACAGGATTCTTGATAACTTTACAGACGACTACGAGAAGTTTCGCTGGAGAGTCCCATTCTAATACGACTCACTAAATGAGACACACCATGTTCAAACTGATTAAGAAGTTAGGCCAACTGCTGGTTCGTATGTACAACGTGGAAGCCAAGCGACTGAACGATGAGGCTCGTAAAGAGGCCACACAGTCACGCGCTCTGGCGATTCGCTCCAACGAACTGGCTGACAGTGCATCCACTAAAGTTACCGAGGCTGCCCGTGTGGCAAACCAAGCTCAAc                                                                                                                                                                                                                     <  1:255211/287‑1 (MQ=255)`  
                                                                                        `agtacaatagattggACTTCTGAAAGGATTCGTGATGATCTACCAGACGACTACGAGAAGTGTCGCAGGGGGGTCCCATTCTAATACAAGCCACTAAAGGGTACACACCAGGTTCAAACTGATGAAGAAGTTAGGCCAACTGCTGGTTCGTATGTACAACGTTGAAGCCAAGCAACTGAACAATTAGGCTCGTAAAGAGGCCACACAGTCACGCGATCTGGCGATTCGCTCCAACGAACTGGCTGACAGTGCATCCACTAAAGTTACCGAGGCTGCCCGTGTGGCAAACCAAGCTCAACAg                                                                                                                                                                                                                   <  1:110502/287‑1 (MQ=255)`  
                                                                                           `ccAACGACACTGACTTCTGACAGGATTCTTGATGACTTTCCAGACGACTACGAGAAGTTTCGCTGGAGAGTCCCATTCTAATACGACTCACTAAAGGAGACACACCATGTTCAAACTGATTAAGAAGTTAGGCCAACTGCTGGTTCGTATGTACAACGTGGAAGCCAAGCGACTGAACGATGAGGCTCGTAAAGAGGCCACACAGTCACGCGCTCTGGCGATTCGCTCCAACGAACTGGCTGACAGTGCATCCACTAAAGTTACCGAGGCTGCCCGTGTGGCAAACCAAGCTCAACAGCtt                                                                                                                                                                                                                <  1:475429/301‑1 (MQ=255)`  
                                                                                                  `cacTGACTTCTGACAGGATTCTTGATGACTTTCCAGACGACTACGAGAAGTTTCGCTGGAGAGTCCCATTCTAATACGACTCACTAAAGGAGACACACCATGTTCAAACTGATTAAGAAGTTAGGCCAACTGCTGGTTCGTATGTACAACGTGGAAGCCAAGCGACTGAACGATGAGGCTCGTAAAGAGGCCACACAGTCACGCGCTCTGGCGATTCGCTCCAACGAACTGGCTGACAGTGCATCCACTAAAGTTACCGAGGCTGCCCGTGTGGCAAACCAAGCTCAACAGCTTTCCAAAt                                                                                                                                                                                                         <  1:176364/301‑1 (MQ=255)`  
                                                                                                    `cTGACTTCTGACAGGATTCTTGATGACTTTCCAGACGACTACGAGAAGTTTCGCTGGAGAGTCCCATTCTAATACGACTCACTAAAGGAGACACACCATGTTCAAACTGATTAAGAAGTTAGGCCAACTGCTGGTTCGTATGTACAACGTGGAAGCCAAGCGACTGAACGATGAGGCTCGTAAAGAGGCCACACAGTCACGCGCTCTGGCGATTCGCGCCAACGAACTGGCTGACAGTGCATCCACTAAAGTTACCGAGGCTGCCCGTGTGGCAAACCAAGCTCAACAGCTTTCCAAATTc                                                                                                                                                                                                       <  1:166733/301‑1 (MQ=255)`  
                                                                                                      `gACTTCTGACAGGATTCTTGATTACTTTCCAGACGACTACGAGAAGTTTCGCTGGAGAGTCCCATTCTAATACGACTCACTAAAGGAGACACACCATGTTCAAACTGATTAAGAAGTTAGGCCAACTGCTTGTTCGTATGTACAACGTGGAAGCCAAGCGACTGAACGATGAGGCTCGTAAAGAGGCCACACAGTCACGCGCTCTGGCGATTCGCTCCAACGAACTGGCTGACAGTGCATCCACTAAAGTTACCGAGGCTGCCCGTGTGGCAAACCAAGCTCAACAGCTTTCCAAATTCtt                                                                                                                                                                                                     <  1:148552/301‑1 (MQ=255)`  
                                                                                                        `cTTCTGACAGGATTCTTGATGACTTTCCAGACGACTACGAGAAGTTTCGCTGGAGAGTCCCATTCTAATACGACTCACTAAAGGAGACACACCATGTTCAAACTGATTAAGAAGTTAGGCCAACTGCTGGTTCGTATGTACAACGTGGAAGCCAAGCGACTGAACGATGATGCTCGTAAAGAGGCCACACAGTCACGCGCTCTGGCGATTCGCTCCAACGAACTGGCTGACAGTGCATCCACTAAAGTTACCGAGGCTGCCCGTGTGGCAAACCAAGCCCAACAGCTTTCCAAATTCtttt                                                                                                                                                                                                   >  1:404919/1‑300 (MQ=255)`  
                                                                                                           `cTGACAGGATTCTTGATGACTTTCCAGACGACGACGAGAAGTTTCGCTGGAGAGTCCCATTCTAATACGACTCACTAAAGGAGACACACCACGTTCAAACTGATTAAGAAGTTAGGCCAACTGCTGGTTCGGATGTACAACGTGGAAGCCAAGCGACTGAACGATGAGGCTAGTAAAGAGGCCACACAGTCACGCGCTCTGGCGATTCGCTCCAACGAACTGGCTGACAGTGCATCCACTAAAGTTACCGAGGCTGCCCGTGTGGCAAACCAAGCTCAACAGCTTTCCAAATTCTTTGAGt                                                                                                                                                                                                <  1:59987/301‑1 (MQ=255)`  
                                                                                                                   `agaattGATGACTTGCCGGACGAATACGAGAAGGTTCGCTGGGGAGTACCAATCTAAGACGACTAACTAAAGGAGACACAACATGTTCAAACTGAGTAAGAAGTTAGGCCAACTGCTGGTTCGTATGTACAACGTGGAAGCCAAGCGACTGGACGATGAGGCTCGTAAAGAGGCCACACAGTCACGCGCTCTGGCGATTCGCTCCAACGAACTGGCTGACAGTGCATCCACTAAAGTTACCGAGGCTGCCCGTGTGGCAAACCAAGCTCAACAGCTTTCCAAATTCTTTGAGTAATAAAAc                                                                                                                                                                                        <  1:42373/297‑1 (MQ=255)`  
                                                                                                                   `aTTCTTGATGACTTTCCAGACGACTACGAGAAGTTTCGCTGGAGAGTCCCATTCTAATACGACTCACTAAAGGAGACACACCATGTTCAAACTGATTAAGAAGTTAGGCCAACTGCTGGTTCGTATGTACAACGTGGAAGCCAAGCGACTGAACGATGAGGCTCGTAAAGAGGCCACACAGTCACGCGCTCTGGCGATTCGCTCCAACGAACTGGCTGACAGTGCATCCACTAAAGTTACCGAGGCTGCCCGTGTGGCAAACCAAGCTAAACAGCTTTCCAAATTCTTTGAGTAATCaaaa                                                                                                                                                                                        >  1:22216/1‑300 (MQ=255)`  
                                                                                                                     `tCTTGATGACTTTCCAGACGACTACGAGAAGTTTCGCTGGAGAGTCCCATTCTAATACGACTCACTAAAGGAGACACACCATGTTCAAACTGATTAAGAAGTTAGGCAAACTGCTGGTTCGTATGTACAACGTGGAAGCCAAGCGACTGAACGATGAGGCTCGTAAAGAGGCCACACAGTCACGCGCTCTGGCGATTCGCTCCAACGAACTGGCTGACAGTGCATCCACTAAAGTTACCGAGGCTGCCCGTGTGGCAAACACAGCTCAACAGCTTTCCAAATTCTTTGAGTAATACAACAg                                                                                                                                                                                      >  1:86970/1‑301 (MQ=255)`  
                                                                                                                      `cTTGATGACTTTCCAGACGACTACGAGAAGTTTCGCTGGAGAGTCCCATTCTAATACGACTCACTAAAGGAGACACACCATGTTCAAACTGATTAAGAAGTTAGGCCAACTGCTGGTTCGTATGTACAACGTGGAAGCCAAGCGACTGAACGATGAGGCTCGTAAAGATGCCACACAGTCACGCGCTCTGGCGATTCGCTCCAACGAACTGGCTGACAGTGCATCCACTAAAGTTACCGAGGCTGCCCGTGTGGCAAACCAAGCTCAACAGCTTTCCAAATTCTTTGAGTAATCAAACacg                                                                                                                                                                                     >  1:456581/1‑299 (MQ=255)`  
                                                                                                                      `cTTGATGACTTTCCAGACGACTACGAGAAGTTTCGCTGGAGAGTCCAATTCTAATACGACTCACTAAAGGAGTCACACCATGTTCAAACTGATTAAGAAGTTCGGCCAACTGCTGGTTCGTATGTACAACGTTGAAGCCAAGCGACTGAACGATGAGGCTCGTAAAGAGGCCACACAGTCACTCGCTCTGGCGATTCGCTCCAACGAACTGGCTGACAGTGCCACCACTAAAGTTTCCGAGGCTGCCCGTGTGGCAAACCAAGCTAAACAGCGTTCCAAATTACTTGAGTAATCAAACAgg                                                                                                                                                                                     >  1:83044/1‑301 (MQ=255)`  
                                                                                                                              `aagagacagcgacTACGAGAATGTTCGCTGGAGAGACCCATTCTAAGCCGACTTACTAAATGAGACACACCATGTTCAAACTGATTAAGAAGTTAGGCCAACTGCTGGTTCGTATGTACAACCTGGAAGCCAAGCGACTGAACGATGAGGCTCGTAAAGAGGCCACACAGTCACGCGCTCTGGCGATTCGCTCCAACTAACTGGCTGACAGTGCATCCACTAAATTTACCGAGGCTGCCCGTGTGGCAAACCAAGCTCAACAGCTTTCCAAATTCTTTGAGTAATCAAACAGGAGAAACCa                                                                                                                                                                             <  1:488113/292‑1 (MQ=255)`  
                                                                                                                                 `tCCAGACTACTACGAGAAGTTTCACTGGAGAGTTCCATTCTAAGACGACTCACTAAAGGATACACACGATGTTCAGACTGAATAAGAAGTTAGGCCAACTTCTGGTTCGTATGTACAACGTGGAAGCCAAGCGACTGAACGATGAGGCTCGTAAATAGGCCACACAGTCACGCGCTCTGGCGATTCGCTCCAACGAACTGGCTGACAGTGCATCCACTAAAGTTACCGAGGCTACCCGTGTGGCAAACCAAGCTCAACAGCTTTCCAAATTCTTTGAGTAATCAAACAGGAGAAACCATTA                                                                                                                                                                          <  1:35814‑M1/301‑1 (MQ=255)`  
                                                                                                                                   `cAGACGACTACGAGAAGTTTCGCTGGAGAGTCCCATTCTAATACGACTCACTAAAGGAGACACACCATGTTCAAACTGATTAAGAAGTTAGGCCAACTGCTGGTTCGTATGTACAACGTGGAAGCCAAGCGACTGAACGATGAGGCTCGTAAAGAGGCCACACAGTCACGCGCTCTGGCGATTCGCTCCAACGAACTGGCTGACAGTGCATCCACTAAAGTTACCGAGGCTGCCCGTGTGGCAAACCAAGCTCAACAGCTTTCCAAATTCTTTGAGTAATCAAACAGGAGAAATCAATATG                                                                                                                                                                        <  1:181651‑M1/301‑1 (MQ=255)`  
                                                                                                                                                      `tCGCTGGAGAGTCCCATTCTAATACGACTCACTAAAGGAGACACACCATGTTCAAACTGATTAAGAAGTTAGGCCAACTGCTGGTTCGTATGTACAACGTGGAAGCCAAGCGACTGAACGATGAGGCTCGTAAAGAGGCTACACAGTCACGCGCTCTGGCGATTCGCTCCAACGAACTGGCTGACAGTGCATCCACTAAAGTTACCGAGGCTGCCCGTGTGGCAAACCAAGCTCAACAGCTTTCCAAATTCTTTGAGTAATCAAACAGTAGAAACCATTATGatcgtttctgacatcgaag                                                                                                                                                     >  1:133414‑M1/1‑282 (MQ=255)`  
                                                                                                                                                       `cGCTGGAGAGTCCCATTCTAATACGACTCACTAAAGGAGACACACCATGTTCAAACTGATTAAGAAGTTAGGCCAACTGCTGGTTCGTATGTACAACGTGGAAGCCAAGAGACTGAACGATGAGGCTCGTAAAGAGGCCACACAGTCACGCGCTCTGGCGATTCGCTCCAACGAACTGGCTGACAGTGCATCCACTAAAGTTACCGAGGCTGCACGTGTGGCAAACCAAGCTCAACAGCTTTCCAAATTCTTTGAGTAATCAAACAGGAGAAACCATTATGatcgtttctgacatcgaagc                                                                                                                                                    <  1:454288‑M1/301‑21 (MQ=255)`  
                                                                                                                                                                     `aTTCTAATACGACTCACTAAAGGAGACACACCATGTTCAAACTGATTAAGAAGTTAGGCCAACTGCTGGTTCTTATTTACAACGTGGAATCCAAGCGACTGAACCATGAGGCTCTTAAATAGGCCACCCAGTCACGCGCTCTTTCTATTCGCTCCCACTATCTGGCTGACATTGCATCCACTATAGTTTCCGAGGCTGCCCGTTTGGCAAACCAAGCTCAACCGCTTTCCATCTTCTTTCAGTAATCAAACAGCAGACATCACTATGatcgtttcttacatcgcagcgaactccctcttat                                                                                                                                      >  1:449758‑M1/1‑267 (MQ=255)`  
                                                                                                                                                                     `aTTCTAATACGACTCACTAAAGGAGACACACCATGTTCAAACTGATTAAGAAGTTAGGCCAACTGCTGGTTCGTATGTACAACGTGGAAGCCAAGCGACTGAACGATGAGGCTCGTAAAGAGGCCACACAGTCACGCGCTCTGGCGATTCGCTCCAACGAACTGGCTGACAGTGCATCCACTAAAGTTACCGAGGCTGCCAGTGTGGCAAACCAAGCTCAACAGCTTTCCAAATTCTTTGAGTAATCAAACAGGAGAAAACATTATGatcgtttctgacatcgaagctaacgccctcttag                                                                                                                                      >  1:349980‑M1/1‑267 (MQ=255)`  
                                                                                                                                                                      `ttCTAATACGACTCACTAAAGGAGACACACCATGTTCAAACTGATTAAGAAGTTAGGCCAACTGCTGGTTCGTATGTACAACGTGGAAGCCAAGCGACTGAACGATGAGGCTCGTAAAGAGGCCACACAGTCACGCGCTCTGGCGATTAGCTCCAACGAACTGGCTGACAGTGCATCCACTAAAGTTACCGAGGCTGCCCGTGTGGCAAACCAAGCTCAACAGCTTTCCAAATTCTTTGAGTAATCAAACAGGAGAAACCATTATGatcgtttctgacatcgaagctaacgccctcttaga                                                                                                                                     >  1:25945‑M1/1‑266 (MQ=255)`  
                                                                                                                                                                      `ttCTAATACGACTCACTAAAGGAGACACACCATGTTCAAACTGATTAAGAAGTTAGGCCAACTGCTGGTTCGTATGTACAACGTGGAAGCCAAGCGACTGAACGATGAGGCTCGTAAAGAGGCAACACAGTCACGCGCTCTGGCGATTCGATCCAACGAACTGGCTGACAGTGCATCCACTAAAGTTACCGAGGCTGCCCGTATGGCAAACCAAGCTCAACAGCTTTCCAAATTCTTTGAGTAATCAAACAGGAGAAACCATGATGatcgtttctgacatcgaagctaacgcccctgtcct                                                                                                                                     >  1:64635‑M1/1‑266 (MQ=255)`  
                                                                                                                                                                       `tCTAATACTACTCACTAAAGGAGACACACCATGTTCAAACTGATTAAGAAGTTAGGCCAACTGCTGGTTCGTATGTACAACGTGGAAGCCAAGCTACTGAACGATGAGGCTCGTAAAGAGGCCACACAGTCACGCGCTCTGGCGATTCGCTCCAACGAACTGGCTGACAGTGCATCCACTAAAGTTACCGAGGCTGCTCGTGTGGCAAACCAAGCTCAACAGCTTTCCAAATTCTTTGAGTAATCGAACAGGAGAAACCATTATGatcgtttctgacatcgaagctaacgccctcttagag                                                                                                                                    <  1:143484‑M1/301‑37 (MQ=255)`  
                                                                                                                                                                       `tCTAATACGGCTCACTAAAGGAGACAACCCATGTTCAAACTGATTAAGAAGTTAGGCCAACTGCTGGGTCGTATGTAAAACGTGGAAGCCAAGCGACTGAACGATGAGGCTCGTAAAGAAGCCACACAGTCACGCGCTCTGGCGATTCGCTCCAACGAACTGGCTGACAGTGCATCCACTAAAGTTACCGAGGCTGCCCGTGTGGCAAACCAAGCTCAACAGCTTTCCAAATTCTTTGAGTAATAAAACAGGAGAAATCAATATGatcgtttctgacatcgaagctaacgacctcttagag                                                                                                                                    <  1:30834‑M1/301‑37 (MQ=255)`  
                                                                                                                                                                       `tCTAATACGACTCACTAAAGGAGAAAAACCATGTTCAAACTGATTAAGAAGTTAGGCCAAATGCTGGTTCGTATGTACAACGTGGAAGCCAAGCGACTGAACGATGAGGCTCGTAAAGAGGCCACACAGTCAAGCGCTCTGGCGATTCGCTCCAACGAACTGGCTGACAGTGCATCCACTAAAGTTACCGAGGCTGCCCGTGTGGCAAACCAAGCTCAACAGCTTTCCAAATTCTTTGAGTAATCAAACAGGAGAAACCATTATGatagtttctgacatcgaagctaacgccctcttagag                                                                                                                                    <  1:294371‑M1/301‑37 (MQ=255)`  
                                                                                                                                                                       `tCTAATACGACTAAATAAAGGAGACAAACCATGTTTAAACGTATTAAGAAGTTAGGCCAACTGCTGGTTCGTATGTACAACGTGGAAGCCAAGAGACTGAACGATGAGGCTCGTAAAGAGGCCACACAGTCACGCGCTCTGGCGATTCGCTCCAACGAACTGGCTGACAGTGCATCCACTAAAGTTACCGAGGCTGCCCGTGTGGCAAACCAAGCTCAACAGCTTTCCAAATTCTTTGAGTAATCAAACAGGAGAAACCATTATGatcgtttctgacatcgaagctaacgccctcttagag                                                                                                                                    <  1:277536‑M1/301‑37 (MQ=255)`  
                                                                                                                                                                       `agatgtgtataagagacagggagacacaccaagttcaaacTGATTAAGAAGTTAGGCCAACTGCTGGTTCGTATGTACAACGTGGAAGCCAAGCGACTGAACGATGAGGCTCGTAAAGAGGCCACACAGTCACGCGCTCTGGCGATTCGCTCCAACGAACTGGCTGACAGTGCATCCACTAAAGTTACCGAGGCTGCCCGTGTGGCAAACCAAGCTCAACAGCTTTCCAAATTCTTTGAGTAATCAAACAGGAGAAACCATTATGatcgtttctgacatcgaagctaacgccctcttagag                                                                                                                                    <  1:398643‑M1/282‑37 (MQ=255)`  
                                                                                                                                                                        `aatgtgtaaaagagacagggagacacaccatgttcaaaCTGATTAAGAAGTTAGGCCAACTGCTGGTTCTTATGTACAACGTGGAAGCCAAGCGACTGAACGATGAGGCTCGTAAAGAGGCCACACAGTCACGCGCTCTGGCGATTCGCTCCAACGAACTGGCTGACAGTGCATCCACTAAAGTTACCGAGGCTGCCCGTGTGGCAAACCAAGCTCAACAGCTTTCCAAATTCTTTGAGTAATC‑AATAGGAGAAATCAATATGatcgtttctgacatcgaagctaacgccatcttagagag                                                                                                                                  <  1:210730‑M1/283‑39 (MQ=255)`  
                                                                                                                                                                               `acagcactaAAGGAGACACACCATGTTCAAACTGATTAAGAAGTTAGGCCAACTGCTGGTTCGTATGTACAACGTGGAAGCCAAGCGACTGAACGATGAGGCTCGTAAAGAGGCCACACAGTCACGCGCTCTGGCGATTCGCTCCAACGAACTGGCTGACAGTGCATCCACTAAAGTTACCGAGGCTGCCCGTGTGGCAAACCAAGCTCAACAGCTTTCCAAATTCTTTGAGTAATCAAACAGGAGAAACCATTATGatcgtttctgacatcgaagccaacgccctcttagagagcgtcac                                                                                                                            <  1:289038‑M1/297‑45 (MQ=255)`  
                                                                                                                                                                                `aCTCACTAAAGGAGACACACCATGTTCAAACTGATTAAGAAGTTAGGCCAACTGCTGGTTCGTATGTACAACGTGGAAGCCAAGCGACTGAACGATGAGGCTCGTAAAGAGGCCACACAGTCACGCGCTCTGGCGATTCGCTCCAACGAACTGGCTGACAGTGCATCCACTAAAGTTACCGAGGCTGCCCGTGTGGCAAACCAAGCTCAACAGCTTTCCAAATTCTTTGAGTAATCAAACAGGAGAAACCATTATGatcgtttctgacatcgaagctaacgccctcttagagagcgtcact                                                                                                                           >  1:376185‑M1/1‑256 (MQ=255)`  
                                                                                                                                                                                   `cACTACAGGAGACACACCATGTTCAAACTGATTAAGAAGTTAGGCCAACTGCTGGTTCGTATGTACAACGTGGAAGCCAAGCGACTGAACGATGAGGCTCGTAAAGAGGCCACACAGTCACGCGCTCTGGCGATTCGCTCCAACGAACTGGCTGACAGTGCATCCACTAAAGTTACCGAGGCTGCCCGTGTGGCAAACCAAGCTCAACAGCTTTCCAAATTCTTTGAGTAATCAAACAGGAGAAACCATTATGatcgtttctgacatcgaagctaacgcactcttcgagagcggcactaaa                                                                                                                        >  1:36282‑M1/1‑253 (MQ=255)`  
                                                                                                                                                                                   `cACTAAAGGAGACACACCATGTTCAAACTGATTAAGAAGTTAGGCCAACTGCTGGTTCGTAGGTACAACGTGGAAGCCAAGCGACTGAACGATGAGGCTCGTAAAGAGGACACACAGTCACGCGCTCTGGCGATTCGCTCCAACGAACTGGCTGACAGTGCATCCACTAAAGTTACCGAGGCTGCCAGTGTGGCAAAACAAGCTCAACAGCTTTCCAAATTCTTTGCGTAATC‑AATAGGAGAAATCAATATGatcgtttctgacatcgaagcaaacgcccacttagagctgtcacttataa                                                                                                                       >  1:130938‑M1/1‑252 (MQ=255)`  
                                                                                                                                                                                    `aagaaaggaGACACAACATGTTAAAACTGATTAAGAAGTTAGGCCAACTGCTGGTTCGTATGTACAACGTGGAAGCCAAGCGACTGAACGATGAGGCTCGTAAAGAGGCCACACAGTCACGCGCTCTGGCGATTCGCTCCAACGAACTGGCTGACAGTGCATCCACTAAAGTTACCGAGGCTGCCCGTGTGGCAAACCAAGCTCAACAGCTTTCCAAATTCTTTGAGTAATCAAACAGGAGAAACCATTATGatcgtttctgacatcgaagctaacgccctcttagagagcgtcactaagt                                                                                                                       <  1:386813‑M1/298‑50 (MQ=255)`  
                                                                                                                                                                                    `aCTAAAGGAGACACACCATGTTCAAACTTATTAAGAAGTTAGGCCAACTGCTGGTTCGTATGTACAACGTGGAAGCCAAGCGACTGAACGATGAGGCTCGTAAAGAGGCCACACAGTCACGCGCTCTGGCGATTCGCTCCAACGAACTGGCTGACAGTGCATCCACTAAAGTTACCGAGGCTGCCCGTGTGGCAAACCAAGCTCAACAGCTTTCCAAATTCTTTGAGTAATCAAACAGGAGAAACCATTATGatcgtttctgacatcgaagctaacgccctcttagagagcgtcactaagt                                                                                                                       <  1:427135‑M1/301‑50 (MQ=255)`  
                                                                                                                                                                                    `aCTAAAGGAAACACACCATGTTCAAACTGATTAATAAGTTAGGCCAACTGCCGGTTCGTATGTACAACGTGGAAGCCAAGCGACTGAACGATGAGGCTCGTAAAGAGGCCACACAGTCACGCGCTCTGGCGATTCGCTCCAACGAACTGGCTGACAGTGCATCTACTAAAGTTACCGAGGCTGCCCGTGTGGCAAACCAAGCTCAACAGCTTTCCAAATTCTTTGAGTAATCAAACAGGAGAAACCATTATGatcgtttctgacatcgaagctaacgccctcttagagagcgtcactaagt                                                                                                                       <  1:235922‑M1/301‑50 (MQ=255)`  
                                                                                                                                                                                     `cTAAAGGAGACACACCATGTTCAAACTGATTAAGAAGTTAGGCCAACTGCTGGTTCGTATGTACAACGTGGAAGCCAAGCGACTGAACGATGAGGCTCGTAAAGAGGCCACAAAGTCACGCGCTCTGGCGATTAGCTCCAACGAACTGGCTGACAGTGCATCCACTAAAGTTACCGAGGCTGCCCGTGTGGCAAACCAAGCTCAACAGCTTTCAAAATTCTTTGAGTAATCAAACAGGAGAAACCATTATGatcgtttctgacatcgaagctaacgccctcttagagagcgtcactaagtt                                                                                                                      >  1:25168‑M1/1‑251 (MQ=255)`  
                                                                                                                                                                                     `atgtgtaaaagatacagtgttcaaactgattaagaAGTGAGGCCAACTGCTGGTTCGTATGTACAACGTGGAAGCCAAGCGACTGAACGATGAGGCTCGTAAAGTGGCTACACAGTCACGCGCTCTGGCGATTCGCTCCAACGAACTGGCTGACAGTGCATCCACTAAAGTTACCGAGGCTGCCCGTGTGGCAAACCAAGCTAAACAGCTTTCCAAATTCTTTGAGTAATC‑AATAGGAGAAATCAATATGatcgtttctgacatcgaagctaacgccctcttagagagcgtcactaagttc                                                                                                                     <  1:480952‑M1/284‑52 (MQ=255)`  
                                                                                                                                                                                       `gtgtataagagacagtgttcaaactgattaaGAAGTTAGGCCAACTGCTGGTTCGTATGTAAAACGTGGAAGCCAAGCGCATGAACGATGAGGCTCGTAAAGAGGCCACACAGTCACGCGCTCTGGCAATTCGCTCCAACGAACTGGCTGACAGTGCATCCACTAAAGTTACCTAGGCTGCCCGTGTGGCAAACCAAGATCAACAGCTTTCCAAATTCTTTGAGTAATCAAACTGGAGAAACCATTATGagcgtttctgacatcgaagctaacgccctcttagagagcgtcactaagttcc                                                                                                                    <  1:74912‑M1/286‑53 (MQ=255)`  
                                                                                                                                                                                       `agaggAGACACACCATGTTCAAACTGATTAAGAAGTTAGGCCAACTGCTGGTTCGTATGTACAACGTGGAAGCCAAGCGACTGAACGATGAGGCTCGTAAAGAGGCCACACAGTCACGCGCTCTGGCGATTCGCTCCAACGAACTGGCTGACAGTGCATCCACTAAAGTTACCGAGGCTGCCCGTGTGGCAAACCAAGCTCAACAGCTTTCCAAATTCTTTGAGTAATCAAACAGGAGAAACCATTATGatcgtttctgacatcgaagctaacgccctattagagagcgtcactaagttcc                                                                                                                    <  1:324492‑M1/299‑53 (MQ=255)`  
                                                                                                                                                                                           `gagaCACACCATGTTCAAACTGATTAAGAAGTTAGGCCAACTGCTGGTTCGTATGTACAACGTGGAAGCCAAGCGACTGAACGATGAGGCTCGTAAAGAGGCCACACAGTCACGCGCTCTGGCGATTCGCTCCAACGAACTGGCTGACAGTGCATCCACTAAAGTTACCGAGGCTGCCCGTGTGGCAAACCAAGCTCAACAGCTTTCCAAATTCTTTGAGTAATCAAACAGGAGAAACCATTATGatcgtttctgacctcgaagctaacgccctcttagagagcgtcactaagttccacct                                                                                                                >  1:101444‑M1/1‑245 (MQ=255)`  
                                                                                                                                                                                                `accccatgTTCAAACTGATTAAGAAGTTAGTCGAACTGCTTGTGCGTATGTACAACGTGGAAGACAAGCGCCTGAACGATGAGGCTCGTAAAGAGGCCACACAGTCACGCGCTCTGGCGATTCGCTCCACCGAACTGGCTGACAGTTCATCCACTAAAGTTACCGAGGCTGCCCGTGTGGCAAACCAAGCTAAACAGCTTTCCAAATTCTTTGAGTAATCAAACAGGAGAAACCATTCTGatcgtttctgacatcgaagctaacgccctcttagagagcgtcactaagttccactgcgggg                                                                                                           <  1:117213‑M1/298‑62 (MQ=255)`  
                                                                                                                                                                                                 `cacCATGTTCAAACTGATTAAGAAGTTAGGCCAACTGCTGGTTCGTATGTACAACGTGGAAGCCAAGCGACTGAACGATGAGGCTCGTAAAGAGGCCACACAGTCACGGGCTATGGCGATTCGCTCCAACGAACTGGCTGACAGTGCATCCACTAAAGTTACCGAGGCTGCCCGTGTGGCAAACCAAGCTCAACAGCTTTACAAATTCTTTGAGTAATCAAACAGGAGAAACCATTATGatcgtttctgacatcgaagctaacgccctcttagagagggtcacaacgttccactgcggggt                                                                                                          >  1:318533‑M1/1‑239 (MQ=255)`  
                                                                                                                                                                                                 `cacCATGTTCAAACTGATTAAGAAGTTAGGCCAACTGCTGGTTCGTATGTACAACGTGGAAGCCAAGCGACTGAACGATGAGGCTCGTAAAGAGGCCACACAGTCACGCGCTCTGGCGATTCGCTCCAACGAACTGGCTGACAGTGCATCCACTAAAGTTACCGAGGCTGCCCGTGTGGCAAACCAAGCTCAACAGCTTTCCAAATTCTTTGAGTAATCAAACAGGAGAAACCATTATGatcgtttctgacatcgaagctaacgccctcttagagagcgtaactaagttccactgcggggt                                                                                                          >  1:41364‑M1/1‑239 (MQ=255)`  
                                                                                                                                                                                                   `ccATGTTCAAACTGATTAAGAAGTTAGGCCAACTGCTGGTTCGTATGTACAACGTGGAAGCCAAGCGACTGAACGATGAGGCTCGTAAAGAGGCCACACAGTCACGCGCTCTGGCGATTCGCTCCAACGAACTGGCTGACAGTGCATCCACTAAAGTTACCGAGGCTGCCCGTGTGGCAAACCAAGCTCAACAGCTTTCCAAATTCTTTGAGTAATCAAACAGGAGAAACCATTATGatcgtttctgacatcgaagctaacgccctcttagagctgtctcttatacacatctccgagccca                                                                                                        >  1:367502‑M1/1‑237 (MQ=255)`  
                                                                                                                                                                                                      `tGTTCAAACTGATTAAGAAGTTAGGCCAACTGCTGGTTCGTATGTACAACGTGGAAGCCAAGCGACTGAACGATGAGGCTCGTAAAGAGGCCACACAGTCACGCGCTCTGGCGATTCGCTCCAACGAACTGGCTGACAGTGCATCCACTAAAGTTACCGAGGCTGCCCGTGTGGCAAACCAAGCTCAACAGCTTTCCAAATTCTTTGAGTAATCAAACAGGAGAAACCATTATGatcgtttctgacatcgaagctaacgccctcttatagagcgtcactaagttccactgcggggttatct                                                                                                     >  1:207035‑M1/1‑234 (MQ=255)`  
                                                                                                                                                                                                      `tGTTCAAACTGATTAAGAAGTTAGGCCAACTGCTGGTTCGTATGTACAACGTGGAAGCCAAGCGACTGAACGATGAGGCTCGTAAAGAGGCCACACAGTCACGCGCTCTGGCGATTCGCTCCAACGAACTGGCTGACAGTGCATCCACTAAAGTTACCGAGGCTGCCCGTGTGGCAAACCAAGCTCAACAGCTTTCCAAATTCTTTGAGTAATCAAACAGGAGAAACAATCATGatcgtttctgacatcgaagctaacgccctcttagagagcgtcactaagttccactgcggggttatac                                                                                                     >  1:465718‑M1/1‑234 (MQ=255)`  
                                                                                                                                                                                                      `tGTTCAAACTGATTAAGAAGTTAGGCCAACTGCTGGTTCGTATGTACAACGTGGAAGCCAAGCGACTGAACGATGAGGCTCATAAAGAGGCCACACAGTCACGCGCTCTGGCGATTCGCTCCAACGAACTGGCTGACAGTGCATCCACTAACGTTACCGAGGCTGCCCGTGTGGCAAACCAAGCTCAACAGCTTTCCAAATTCTTTGAGTAATCAAACAAGAGAAACCATGATGatcgtttctgacatcgaagctaacgacctcgtagagagcgtaactaagttccactgctgggtgatcc                                                                                                     >  1:313320‑M1/1‑234 (MQ=255)`  
                                                                                                                                                                                                        `gggctaggagatgtgtagaagagacagcttctggttcgtatgtacaacgtggaagccaagcgaCTGAACGATGAGGCTCGTAAAGAGGCCACACAGTCACGCGCTCTGTCGATTCGATCCAACGAACTGGCTGACGGTGCATCCACTAAAGTTACCGAGGCTGCCCGTGTGGCAAACCAAGCTCAACAGCTTTCCAAATTCTTTGAGTAATCAAACAGGAGAAATCAATATGatcgtttctgacatcgaagctaacgccctcttagagagcgtcactaagttccactgcggggttatctac                                                                                                   <  1:506696‑M1/271‑70 (MQ=255)`  
                                                                                                                                                                                                           `aaaCTGATTAATGAGTTAGGCAAACTGATGGTTCGGATGTACAACGTGGAAGCCAAGCGACGGAACGATGAGGCTCGTAAAGAGGCCACACAGTCACGCGCTGTGGCGATTAGATCCAACGAACTGGCTGACAGTGCATCCACTAAAGTTACCGAGGCTGCCCGTGTGGCAAACCAAGCTCAACAGCTTTCCAAATTCTTTGAGTAATCAAACAGGAGAAACCAATATGatcgtttctgacatcgaagctaacgccctcttagagagcgtcactaagttccactgcggggttatctacgac                                                                                                <  1:5271‑M1/301‑73 (MQ=255)`  
                                                                                                                                                                                                            `aaCTGATTAAGAAGGTAGGCCAACTGCTGGTTCGGATGTACAACGTGGAAGCCAAGCGACTGAACGATGAGGCTCGTAAAGAGGCCACACAGTCACGCGCTCTGGCGATTCGCTCCAACGAACTGGCTGACAGTGCATCCACTAAAGTTACCGAGGCTGACCGTGTTGCAAACCAAGCTCAACAGCTTTCCAAATTCTTTGAGTAATCAAACAGGAGAAACCATTATGatcgtttctggcatcgaagctaacgccctcttagagagcgtcactaagttccactgcggggttatctacgact                                                                                               <  1:44090‑M1/301‑74 (MQ=255)`  
                                                                                                                                                                                                              `cTGATTAAGAAGTTAGGCCAACTGCTGGTTCGTATGTACAACGTGGAAGCCAAGCGACTGAACGATGAGGCTCGTAAAGAGGCCACACAGTCACGCGCTCTGGCGATTCGCTCCAACGAACTGGCTGACAGTGCATCCACTAAAGTTACCGAGGCTGCCCGTGTGGCAAACCAAGCTCAACAGCTTTCCAAATTCTTTGAGTAATCAAACAGGAGAAACCATTATGatcgtttctgacatcgaagctaacgccctcttagagagcgtcactaagttccactgcggggttatctacgacctg                                                                                             >  1:10714‑M1/1‑226 (MQ=255)`  
                                                                                                                                                                                                               `tgatgaAGAAGTTAGGCCAACTGCTGGTTCGTATGTACAACGTGGAAGCCAAGCGACTGAACGATGAGGCGCGTAAAGAGTCCACACAGTCACGCGCTCTGGCGATTCGCGCCAACGAACTGGCTGACAGTGCATCCACTAAAGTTACCGAGGCTGCCCGTGTGGCAAACCAAGCTCAACAGCTTTCCAAAGTCTTTGAGTAATCAAACAGGAGAAA‑CATTATGatcgtttctgacatcgaagctaactccctcttagagagcgtcactaagttccactgcggggttatctacgactactc                                                                                           <  1:139594‑M1/301‑78 (MQ=255)`  
                                                                                                                                                                                                                `gATTAAGAAGTTAGGCCAACTGCTGGTTCGTATGTACAACGTGGAAGCCAAGCGACTGAACGATGAGGCTCGTAAAGAGGCCACACAGTCACGCGCTCTGGCGATTCGCTCCAACGAACTGGCTGACAGTGCATCCACTAAAGTTACCGAGGCTGCCCGTGTGGCAAACCAAGCTCAACAGCTTTCCAAATTCTTTGAGTAATCAAACAGGAGAAACCATTATGatcgtttctgacatcgaagctaacgccctcttagagagcgtcactaagttccactgcggggttatctacgactactc                                                                                           >  1:461202‑M1/1‑224 (MQ=255)`  
                                                                                                                                                                                                                `gATTAAGAAGTTAGGCCAAATGCTGGTTCGTATGTACAACACGGAAGCCAAGCGACTGGACGATGAGACGCGTAAAGAGGCCACACAGTCACGCGCTCTGGCGATTCGCTCCAACGAACTGGCTGAAAGTGGATCCACTAAAGTTACCGAGGCTGGCCGTGTGGCAAACAAAGTTAATCAGCGTTCCAAATTCTTTGAGTAATAAAACAGGAGAAATCAATATGatcgtttctgacatcgaagctaacgccctcttagagagcgtcactaagttccactgcggggttatctacgactactc                                                                                           <  1:333888‑M1/301‑78 (MQ=255)`  
                                                                                                                                                                                                                     `agaagTAAGGCCAACTGCGGGTTCGTATGTACAACGTGGAAGCCAAGCGACTGAACGATGAGGCTCGTAACGAGGCCACACAGTCACGCGCTCTGGCGATTCGCTCCAACGAACTGGCTGACAGTGCATCCACTAAAGTTACCGAGGCTGCCCGTGTGGCAAACCAAGCTCAACAGCTTTCCAAATTCTTTGAGTAATCAAACAGGAGAAATCAATATGatcgtttctgacatcgaagctaacgccctcttagagagcgtcactaagttccactgcggggttagagacgactactcaaccg                                                                                      <  1:203221‑M1/301‑83 (MQ=255)`  
                                                                                                                                                                                                                      `gaagTTAGGCCAACTGCTGGTTCGTATGTACAACGTGGAAGCCAAGCGCCTGAACGATGAGGCTCGTAAAGAGGCCACACAGTCACGCGCTCTGGCGATTCGCTCCAACGAACTGGCTGACAGTGCATCCACTAAAGTTACCGAGGCTGCCCGTGTGGCAAACCAAGCTCAACAGCTTTCCAAATTCTTTGAGTAATCAAACAGGAGAAACCATTATGatcgtttctgacatcgaagctaacgccatcttagagagcgtcactaagttccactgcggggttatctacgactactccaccgc                                                                                     <  1:431898‑M1/301‑84 (MQ=255)`  
                                                                                                                                                                                                                            `aGGCCAACGGCTGGTTCGTATGTACAACGTGGAAGCCAATCGACTGAACGATTAGGCTCGTAAAGAGGCCACACAGTCACGCGCTCTGGCGATTCGCTCCAACGAACTGGCTGACAGTGCATCCACTAAAGTTCCCGAGGCTGCCCGTGTGGCAAACCAAGCTCAACAGCTTTCCAAATTCTTTGAGTAATCAAACAGGAGAAACCATTATGatcgtttctgacatcgaagctaacgccctcttagagagcgtcactaagttccactgcggggttatctacgactactccaccgctgagta                                                                               <  1:152200‑M1/301‑90 (MQ=255)`  
                                                                                                                                                                                                                              `gCCAACTGCTGGTTCGTATGTACAACGTGGAAGCCAAGCGACTGAACGATGAGGCTCGTAAAGAGGCCACACAGTCACGCGCTCTGGCGATTCGCTCCAACGAACTGGCTGACAGTGCATCCACTAAAGTTACCGAGGCTGCCCGTGTGGCAAACCAAGCTCAACAGCTTTCCAAATTCTTTGAGTAATCAAACAGGAGAAACCATTATGatcgtttctgacatcgaagctaacgccctcttagagagcgtcactaagttccactgcggggttatctacgactactgtctcttatccacat                                                                             >  1:444406‑M1/1‑210 (MQ=255)`  
                                                                                                                                                                                                                                   `ctgctgGTTCGTATGTAAAACGTGGAAGCCAAGCGACTGAACGATGAGGCTCGTAAAGAGGCCACACAGTCACGCGCTCTGTCGATTCGCTCCAACGAACTGGCTGAGAGTGCATACACTAAAGTTACCGAGGCTGCTCGGGTGGCAAACCAAGCTCAACAGCTTTCCAAATTCTTTGAGTAATCAAACAGGAGAAACCATTATGatcgtttctgacatcgaagctaacgccctcttagagagcgtcactaagttccactgcgggtttatctacgactactccaccgctgagtacgtaagc                                                                        <  1:246841‑M1/301‑97 (MQ=255)`  
                                                                                                                                                                                                                                         `gTTCGTATGTACAACGTGGAAGCCAAGCGACTGAACGATGAGGCTCGTAAAGAGGCCACACAGTCACGCGCTCTGGCTATTCGCTCCAACGAACTCGCTGACAGTGCATCCACTAAAGTTACCGAGGCTGCCCGTGTGGCAAACCAAGCTCAACAGCTTTCCAAATTCTTTGAGTAATCACACAGGAGAAACCATTATGatcgtttctgacatccaagctaaccccctcttagcgagcgtcactaagttccactgcggggttatctactactactccccctctgagtacgtaagctaccgc                                                                  >  1:35354‑M1/1‑199 (MQ=255)`  
                                                                                                                                                                                                                                              `taggtacAACGTGGAAGCCAAGCGACTGAACGATAAGGCTCGTAAAGATGCCACACAGTCACGCGCTCTGGCGATTCGCTCCAACGAACTGGCTGAACGTGCATCCACTAAACTTACCGAGGCTGCCCGTGTGGCAAACCAAGCTCAACAGCTTTCCAAATTCTTTGAGCAATCAAACAGGAGAAACCATGATGatcgtttctgacatcgaagctaacgccctcttagagatcgtcactaagttccactgcggggttatctacgactactccaccgctgagtacgtaagctaccgtccgag                                                             <  1:156532‑M1/298‑108 (MQ=255)`  
                                                                                                                                                                                                                                                `tGTACAACGTGGAAGCCAAGCGACTGAACGATGAGGCTCGTAAAGAGGCCACACAGTCACGCGCTCTGGCGATTCGCTCCAACGAACTGGCTGACAGTGCATCCACTAAAGTTACCGAGGCTGCCCGTGTGGCAAACCAAGCTCAACAGCTTTCCAAATTCTTTGAGTAATCAAACAGGAGAAACCATTATGatcgtttctgacatcgaagctaacgccctcttagagagcgtcactaagttccactgcggggttatctacgactactccaccgctgagtacgtaagctacctgtctctta                                                           >  1:263304‑M1/1‑192 (MQ=255)`  
                                                                                                                                                                                                                                                 `gTACAACGTGGAAGCCAAGCGACTGAACGATGAGGCTCGTAAAGAGGCCACACAGTCACGCGCTCTGGCGATTCGCTCCAACGAACTGGCTGACAGTGCATCCACTAAAGTTACCGAGGCTGCCCGTGTGGCAAACCCAGCTCAACAGCTTTCCAAATTCTTTGAGTAATCAAACAGGAGAAACCATTATGatcgtttctgacatcgaagctaacgccctcttagagagcgtcactaagttccactgcggggttatctacgactactccaccgctgagtacgtaagctaccgtccgagtga                                                          >  1:232882‑M1/1‑191 (MQ=255)`  
                                                                                                                                                                                                                                                 `gTACAACGTGGAAGCCAAGCGACTGAACGATGAGGCTCGTAAAGAGGCCACACAGTCACGCGCTCTGGCGATTCGCTCCAACGAACTGGCTGACAGTGCATCCACTAAAGTTACCGAGGCTGCCCGTGTGGCAAACCAAGCTCAACAGCTTTCCAAATTCTTTGAGTAATCAAACAGGAGAAACCATTATGatcgtttctgacatcgaagctaacgccctcttagagagcgtcactaagttccactgcgtggttatctacgactactccaccgcttagtacgtaagctaccgtccgagtga                                                          >  1:56177‑M1/1‑191 (MQ=255)`  
                                                                                                                                                                                                                                                    `cAACGTTGAAGCCAAGCGACTGAACGATGAGGCTCGTAAAGAGGCCACACAGTCACGCGCTCTGGCGATTCGCTCCAACGAACTGGCTGACAGTGCATCCACTAAAGTTCCCGAGGCTGCCCGTGTGGCAAACCAAGCTCAACAGCTTTCCAAATTCTTTGAGTAATCAAACAGGCGAAACCATTATGatcgtttctgacatcgaagctaactccctcttagagcgcgtcactacgttccactccgtggttatctacgcctactccaccgctgagtacgtagcctcccgtccgagttcctt                                                       >  1:282575‑M1/1‑188 (MQ=255)`  
                                                                                                                                                                                                                                                        `gTGGAAGCCAAGCGACTGAACGATGAGGCTCGTAAAGAGGCCACACAGTCACGCGCTCTGGCGATTCGCTCCAACGAACTGGCTGACAGTGCATCCACTAAAGTTACCGAGGCTGCCCGTGTGGCAAACCAAGCTCAACAGCTTTCCAAATTCTTTGAGTAATCAAACAGGAGAAACCATTATGatcgtttctgacatcgaagctaacgccctcttagagagcgtcactaagttccactgcggggttatctacgactactccaccgctgagtacgtaagctaccgtccgagtgctgtctct                                                   >  1:380937‑M1/1‑184 (MQ=255)`  
                                                                                                                                                                                                                                                          `ggAAGCAAAGCGACTGAACGATGAGGCTCGTAAAGAGGCCACACAGTCACGCGCTCTGGCGATTCGCTCCAACGAACTGGCTGACAGTGCATCCACTAAAGTTACCGAGGCTGCACGTGTGGCAAACCAAGCTCAACAGCTTTCCAAATTCTTTGAGTAATCAAACAGGAGAAATCAATATGatcgtttctgacatcgaagctaacgccctcttagagagcgtcactaagttccactgcggggttatctacgactactccaccgctgagtacgtaagctaccgtcccagtgacttcggtgc                                                 <  1:395065‑M1/301‑120 (MQ=255)`  
                                                                                                                                                                                                                                                           `gAAGCCAAGCGACTGAACGATGAGGATCGTAAAGAGGCAACACAGTAACGCGCTCTGGCGATTCGCTCCAACGAACTGGCTGACAGTGCATCCACTAAAGTTACCGAGGCTGCCCGTGTGGCAAACCAAGCTCAACAGCTTTCCAAATTCTTTGAGTAATCATACAGGAGAAACCATTATGatcgtttctgacatcgaagctaacgccctcttagagagagtcactaagttccactgcggggttatctacgactactccaccgctgagtacgtaagctaccgtccgagtgacttcggtgcg                                                <  1:114571‑M1/301‑121 (MQ=255)`  
                                                                                                                                                                                                                                                              `ataagagacaggaacgataaggcTCGTAAAGAGGCCACACAGTCACGCGCTCTGGCGATTCGCTCCAATGAACTGGCTGACAGTGCAACCACTAAAGTTACCGAGGCTGCCCGTGTGGCAAACCAAGCTCAACAGCTTTCCAAATTCTGTGAGTAATCAAACAGGAGAAACCATTATGatcgtttctgacatcgaagctaacgccctcttagagagcgtcactaagttccactgcggggttatctacgactactccaccgcttagtacgtaagctaccgtccgagtgacttcggtgcgtat                                             <  1:495031‑M1/290‑124 (MQ=255)`  
                                                                                                                                                                                                                                                               `ccAAGTGACTGAACGATGAGGCTCGTAAAGAGGCCACACAGTCACGCGATCTGGCGATTCGCTCCAACGAACTGGCTGACAGTGCATCCACTAAAGTTACCGAGGCTGCCCGTGTGGCAAACCAAGCTCAACAGCTTTCCAAATTCTTTGAGTAATCAAACAGGAGAAATCAATATGatcgtttctgacatcgaagataacgccctcttagagagagtcactaagttccactgcggggttatctacgactactccaccgctgagtacgtaagctaccgtccgagtgccgtcggtgcgtatt                                            >  1:121147‑M1/1‑177 (MQ=255)`  
                                                                                                                                                                                                                                                               `ccAAGCGACTGAACGATGAGGCTCGTAAAGAGGCCACACAGTCACGCGCTCTGGCGATTCGCTCCAACGAACTGGCTGACAGTGCATCCACTAAAGTTACCGAGGCTGCCCGTGTGGCAAACCAAGCTCAACAGCTTTCCAAATTCTTTGAGTAATCAAACAGGAGAAATCAATATGatcgtttctgacatcgaagctaacgccatcttagagagcgtcactaagttccactgcggggttatctactactactacaccgctgagtacgtaagctaccgtccgagtgccttcggtgcgtatc                                            >  1:328243‑M1/1‑177 (MQ=255)`  
                                                                                                                                                                                                                                                                      `aCTGAACGATGAGGCTCGTAAAGAGGCCACACAGTCACGCGCTCTGGCGATTCGCTCCAACGAACTGGCTGACAGTGCATCCACTAAAGTTACCGAGGATGCCCGTGTGGCAAACCAAGCTCAACAGCTTTCCAAATTCTTTGAGTAATCAAACAGGAGAAACCATTATGatcgtttctgacatcgaagctaacgccctcttagagagcgtcactaagttccactgcggggttatctacgactactccaccgctgagtacgtaagataccgtccgagtgactctgtctcttatacacatct                                     >  1:409043‑M1/1‑170 (MQ=255)`  
                                                                                                                                                                                                                                                                                    `cTCGTAAAGAGGCCACACAGTCACGCGCTCTGGCGATTCGCTCCAACGAACTGGCTGACAGTGCATCCACTAAAGTTACCGAGGCTGCCCGTGTGGCAAACCAAGCTCAACAGCTTTCCAAATTCTTTGAGTAATCAAACAGGAGAAATCAATATGatcgtttctgacatcgaagctaacgccctcttagagagcgtcactaagttccactgcggggttatctacgactactccaccgctgagtacgtaagctaccgtccgagtgacttcggtgcgtatctggatgcgctggatgccgaag                       >  1:269318‑M1/1‑156 (MQ=255)`  
                                                                                                                                                                                                                                                                                              `ggCCACACAGTCACGCGCTCTGGCGATTCGCTCCAACGAACTGGCTGACAGTGCATCCACTAAAGTTACCGAGGCTGCCCGTGTGGCAAACCAAGCTCAACAGCTTTCCAAATTCTTTGAGTAATCAAACAGGAGAAACCATTATGatcgtttctgacatcgaagctaacgccctcttagagagcgtcactaagttccactgcggggttatctacgactactccaccgctgagtccgtaagctcccgtccgagtgacttcggtgcgtatctggatgcgctggaagccgagtttgcacgagg             >  1:304715‑M1/1‑146 (MQ=255)`  
                                                                                                                                                                                                                                                                                               `gCCACACAGTCACGCGCTCTGGCGATTCGCTCCAACGAACTGGCTGACAGTGCATCCACTAAAGTTACCGAGGCTGCCCGTGTGGCAAACCAAGCTCAACAGCTTTCCAAATTCTTTGAGTAATCAAACAGGAGAAACCATTATGatcgtttctgacatcgaagctaacgccctcttagagagcgtcactacgttccactgcggggttatctacgactactccaccgctgagtacgtaagctaccgtccgagttacttcgttgcgtctctggatgcgctggaagcctgtctcttatacaca            >  1:339259‑M1/1‑145 (MQ=255)`  
                                                                                                                                                                                                                                                                                                       `gTCACGCGCTCTGGCGATTCGCTCCAACGAACTGGCTGACAGTGCATCCACTAAAGTTACAGAGGCTGCCCGTGTGGCAAACCAAGCTCAACAGCTTTCCAAATTCTTTGAGTAATCAAACAGGAGAAATCAATATGatcgtttctgacatcgaagctaacgccatcttcgagagcgtcactaagttccactgcggggttatctacgactactcaaccgctgagtacttaagctaccgtccgagtgacttcggtgcgtatctggatgcgctggaagccgaggttgaacgaggcggtcttat    >  1:344868‑M1/1‑137 (MQ=255)`  
                                                                                                                                                                                                                                                                                                         `cccgcgctCTGGCGATTCGCTCCAACGAACTGGCTGACAGTGCATCCACTAAAGTTACCGAGGCTGCCCGTGTGGCAAACCAAGCTCAACAGCTTTCCAAATTCTTTGAGTAATCAAACAGGAGAAATCATTATGatcgtttctgacatcgaagctaacgccctcttagagagcgtcactaagttccactgcggggttatctacgactactccaccgctgagtacgtaagctaccgtccgagtgacttcggtgcgtatctggatgctctggaagccgaggttgcacgaggaggtcttattg  <  1:502032‑M1/299‑167 (MQ=255)`  
                                                                                                                                                                                                                                                                                                         `cACGCGCTCTGGCGATTCGCTCCAACGAACTGGCTGACAGTGCATCCACTAAAGTTACCGAGGCTGCCCGTGTGGCAAACCAAGCTAAACAGCTTTCCAAATTCTTTGAGTAATCAAACAGGAGAAACCATTATGatcgtttctgacatcgaagctaacgccctcttagagagcgtcactaagttccactgcggggttatctacgactactccaccgctgagtacgtaagctaccgtccgagtgacttcggtgcgtatctggatgcgctggaagccgaggttgcacgaggcggtcttattg  <  1:159239‑M1/301‑167 (MQ=255)`  
                                                                                                                                                                                                                                                                                                         `cACGCGCTCTGGCGATTCGCTCCAACGAACGGGCTGACAGTGCATCCACTAAAGTTACCGAGGCTGCCCGTGTGGCAAACCAAGCTCAACAGCTTGCCAAATTCTTTGAGTAATCAAACAGGAGAAACCATTATGatcgtttctgacatcgaagctaacgccctcttagcgagcgtcactaagttccactgcggggttatctacgactactccaccgctgagtacgtaagctaccgtccgagtgacttcggtgcgtatctggatgcgctggaagccgaggttgcacgaggcggtcttattg  <  1:78936‑M1/301‑167 (MQ=255)`  

                                                                                                                                                                                                                                                                                                            `|`                                                                                                                                                                                                                                                                                                           
`GGCTACATGGAATACAACAAGGAAACCGGATGGCTTGAACCATCAAGTTACTCAGGGGAAGAAGAGTCACACTCAGAGTCAACAGACTGGTCCAACGACACTGACTTCTGACAGGATTCTTGATGACTTTCCAGACGACTACGAGAAGTTTCGCTGGAGAGTCCCATTCTAATACGACTCACTAAAGGAGACACACCATGTTCAAACTGATTAAGAAGTTAGGCCAACTGCTGGTTCGTATGTACAACGTGGAAGCCAAGCGACTGAACGATGAGGCTCGTAAAGAGGCCACACAGTCACGCGCTCTGGCGATTCGCTCCAACGAACTGGCTGACAGTGCATCCACTAAAGTTACCGAGGCTGCCCGTGTGGCAAACCAAGCTCAACAGCTTTCCAAATTCTTTGAGTAATCAAACAGGAGAAACCATTATGTCTAACGTAGCTGAAACTATCCGTCTATCCGATACAGCTGACCAGTGGAACCGTCGAGTCCACATCAACGTTCGCAACGGTAAGGCGACTATGGTTTACCGCTGGAAGGACTCTAAGTCCTCTAAGAATCACACTCAGCGTATGACGTTGACAGATGAGCAAGCAC  >  NC_001604.1/13155‑13752`

  

Alignment Legend

Aligned base mismatch/match (shaded by quality score): `ATCG/ATCG` `< 3 ≤` `ATCG/ATCG` `< 9 ≤` `ATCG/ATCG` `< 17 ≤` `ATCG/ATCG` `< 32 ≤` `ATCG/ATCG` `< 38 ≤` `ATCG/ATCG`

Unaligned base: `atcg`    Masked matching base: `atcg`    Alignment gap: `‑`    Deleted base: `‑`

Reads not counted as support for junction

read\_name Not counted due to insufficient overlap past the breakpoint.

read\_name Not counted due to not crossing MOB target site duplication.
